# Supplementary material for: Supramolecular Functionalisation of B/N Co-Doped Carbon Nano-Onions for Novel Nanocarrier Systems
Source: Materials (Basel). 2022 Aug 30;15(17):5987. doi: 10.3390/ma15175987 (PMC9456768; doi:10.3390/ma15175987)
Supplement: Supplementary file 1 [file materials-15-05987-s001.zip › materials-1846364-supplementary.pdf]

materials

MDPI

Article

Supramolecular functionalisation of B/N co-doped carbon nano-onions for novel nanocarrier systems

Hugh Mohan [1], Valeria Bincoletto [2], Silvia Arpicco [2] and Silvia Giordani [2,*]

[1] School of Chemical Sciences, Dublin City University, Glasnevin, Dublin 9, Ireland
[2] Department of Drug Science and Technology, University of Torino, Via P. Giuria 9, Torino, Italy
* Correspondence: silvia.giordani@dcu.ie

Supplementary Material

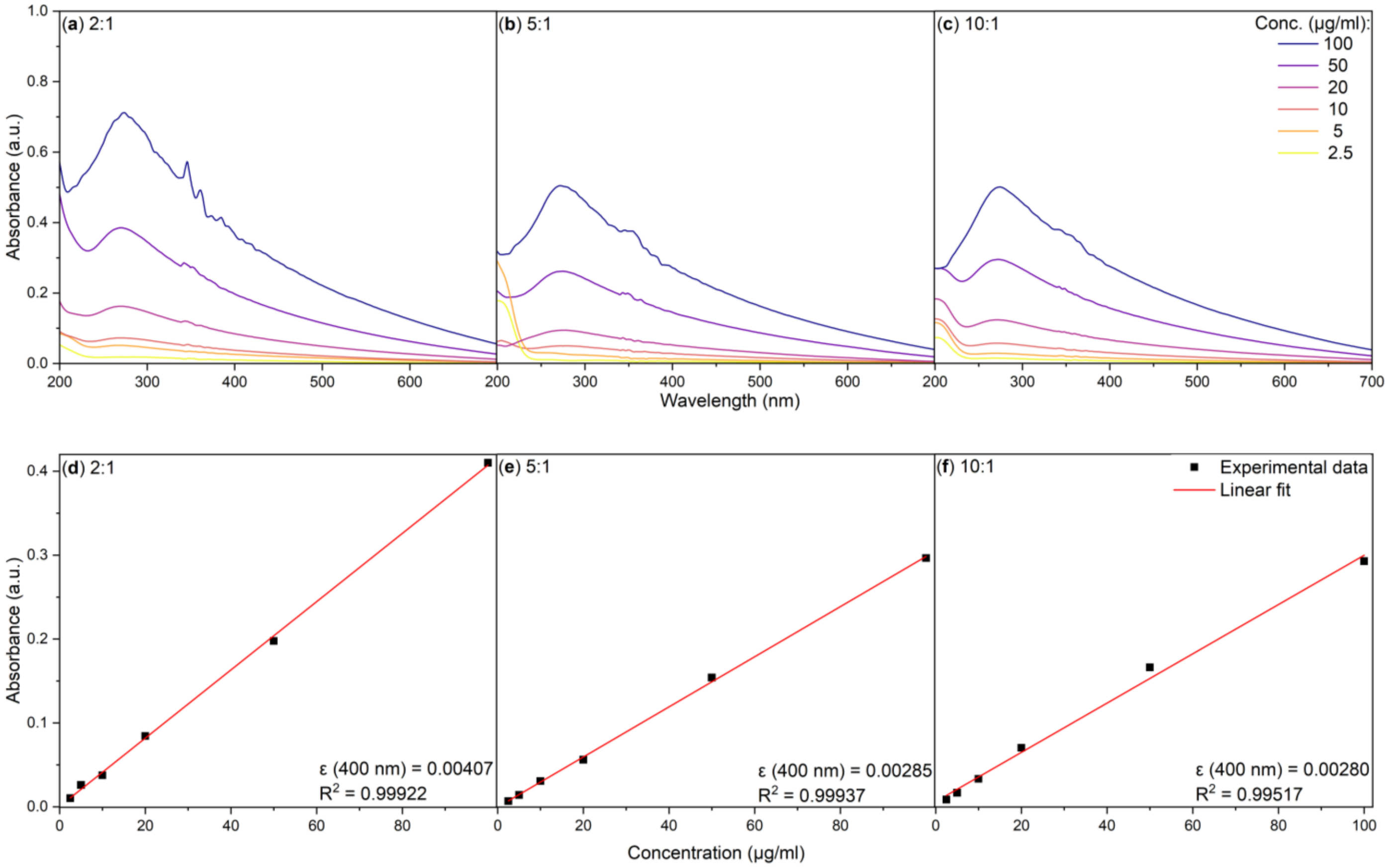

Figure S1. UV-Vis absorption spectra of (**a**) 2:1 BN-CNO/HA-DMPE, (**b**) 5:1 BN-CNO/HA-DMPE and, (**c**) 10:1 BN-CNO/HA-DMPE at different concentrations in deionised water; (**d**) – (**f**) corresponding absorbance vs concentration plots with molar extinction coefficients calculated at 400 nm.

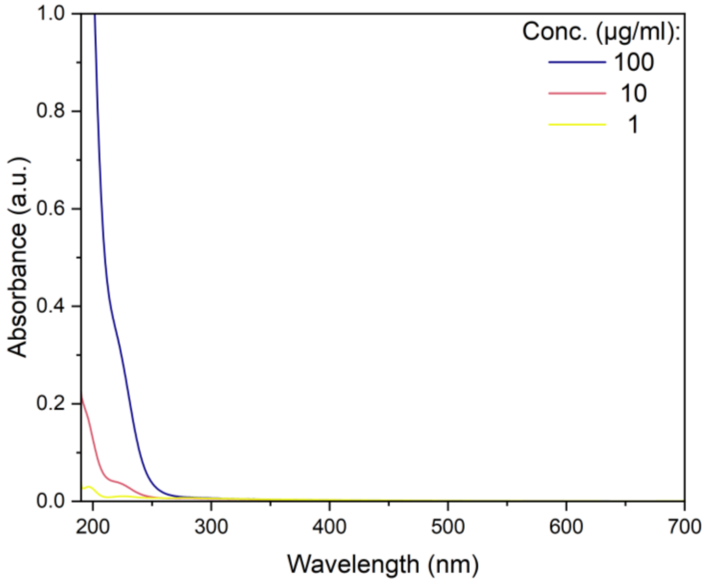

Figure S2. UV-Vis absorption spectrum of HA-DMPE in deionised water at various concentrations.
